# Case Report: Three novel pathogenic *ABCC2* mutations identified in two patients with Dubin–Johnson syndrome

**DOI:** 10.3389/fgene.2022.895247

**Published:** 2022-08-25

**Authors:** Chenyu Zhao, Xiaoliu Shi, Yonghong Zhang, Hui Huang

**Affiliations:** ^1^ Department of Gastroenterology, The Second Xiangya Hospital, Central South University, Changsha, China; ^2^ Department of Medical Genetics, The Second Xiangya Hospital, Central South University, Changsha, China; ^3^ Department of Infectious Diseases, The Second Xiangya Hospital, Central South University, Changsha, China

**Keywords:** Dubin–Johnson syndrome, ABCC2, multidrug resistance-associated protein 2, mutation, hyperbilirubinemia

## Abstract

**Background:** Dubin–Johnson syndrome (DJS) is a rare autosomal recessive genetic disease which is caused by mutations in the *ABCC2* gene; it is characterized by chronic hyperbilirubinemia. Here, we report two pedigrees affected with DJS which were caused by three novel pathogenic *ABCC2* mutations.

**Case summary:** The two patients exhibited intermittent low-grade, predominantly conjugated hyperbilirubinemia and showed no other abnormalities. They were diagnosed clinically with DJS. Three novel pathogenic *ABCC2* mutations—c.2980delA, c.1834C>T, and c.4465_4473delinsGGCCCACAG—were identified by whole-exome sequencing. These mutations could be responsible for DJS in the two pedigrees. The genetic test confirmed the diagnosis of DJS.

**Conclusion:** These results contributed to the genetic diagnosis of the two patients with DJS and expanded the variant database for the *ABCC2* gene.

## Introduction

Dubin–Johnson syndrome (DJS, OMIM*237500) is an autosomal recessive genetic disease which is characterized by intermittent, predominantly conjugated hyperbilirubinemia and liver pigmentation. DJS has no other features of hepatobiliary disorder. First described by Dubin and Johnson (1954), It is considered a rare disorder worldwide, although its incidence among Sephardic Jews has been reported as approximately 1/3000 (Shani et al., 1970). Pathogenic mutations in the adenosine triphosphate-binding cassette subfamily C member 2 (*ABCC2*) gene were first discovered to cause DJS in 1997 (Paulusma et al., 1997). The multidrug resistance-associated protein 2 (MRP2) encoded by the *ABCC2* gene is principally located in the hepatocyte canalicular membrane. Loss-of-function variants in the *ABCC2* gene disturb the secretion of conjugated bilirubin from hepatocytes. DJS does not affect life expectancy and usually requires no treatment (Memon et al., 2016). To date, 127 mutations in *ABCC2* have been described in the Human Gene Mutation Database (HGMD; http://www.hgmd.cf.ac.uk/ac/index. php). In the present study, we reported two patients with DJS and identified three novel disease-causing mutations: c.2980delA, c.1834C>T and c.4465_4473delinsGGCCCACAG, in the *ABCC2* gene (NM_000392).

## Case description

**FIGURE 1:**
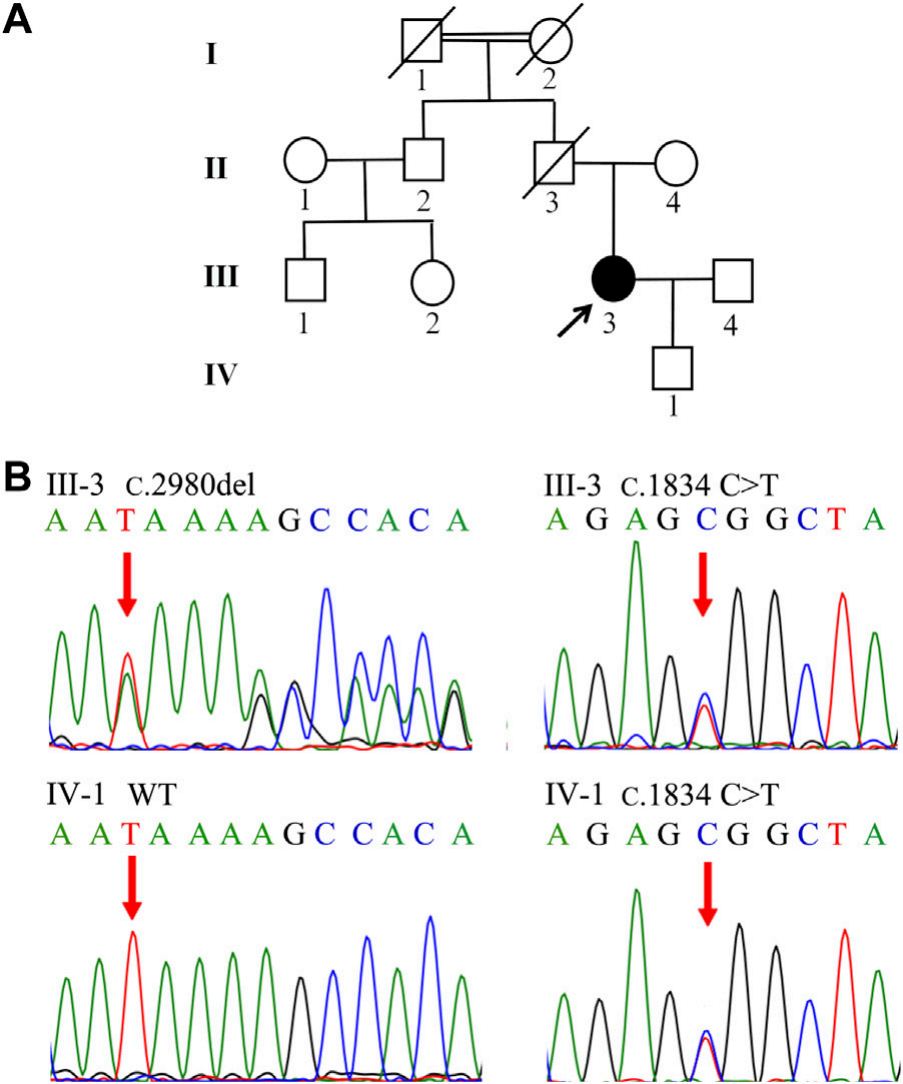
Family tree and genetic analysis of Family 1. **(A)** Proband III-3 was diagnosed with DJS. **(B)** Sanger sequencing revealed a compound heterozygous (c.2980delA and c.1834 C>T) *ABCC2* mutation in III-3. IV-1 shared the heterozygous c.1834 C>T variant.

In Family 1, a 29-year-old woman (Patient 1) was born to nonconsanguineous parents although her grandparents were cousins (Figure 1A). She was affected with low-grade predominantly conjugated hyperbilirubinemia for more than 6 years. This patient was tested for hyperbilirubinemia in a health physical examination in 2015. There were no other significant abnormalities in her liver function test. She received no other medical treatment then. During her pregnancy, her jaundice was markedly aggravated. She gave birth on 18 August 2018. The obstetrician used reduced glutathione (1.2 g/d, from 16 August 2018 to 20 August 2018) and ursodeoxycholic acid (500 mg/d, from 20 August 2018 to 31 August 2018) to control her jaundice. Her clinical jaundice gradually disappeared after childbirth in 2018. She took oral contraceptives from January 2021 and presented with recurrent mild jaundice again. The jaundice was in remission after she stopped taking oral contraceptives; she was never affected with pruritus. The patient had a previously free medical history. When she came to us as a medical genetic outpatient in July 2021, the physical examination revealed mild splenomegaly. The timeline with relevant data for Patient 1 is shown in Supplementary Figure S1. During the patient’s pregnancy, her level of total bilirubin (TBIL) ranged from 43.1 μmol/L to 82.8 μmol/L (normal range: 3.4–17.1 μmol/L). Her direct bilirubin (DBIL) level ranged from 25.2 μmol/L to 65.1 μmol/L (normal range: 0–6 μmol/L). The ratio of DBIL/TBIL was always greater than 50%. There were no abnormalities in other liver function indexes. She was serologically negative for hepatitis A, B, C, and E viruses. There were no abnormalities in coagulation function, autoimmune liver disease-associated antibodies, immunoglobulin G, or serum ceruloplasmin testing. The abdominal ultrasound examination revealed her portal vein diameter to be about 10 mm and the portal venous maximum velocity was 18 cm/s. The thickness of her spleen was about 49 mm and its inferior margin was about 20 mm below the costal margin. A hepatic hemangioma was also detected by the ultrasound examination (Supplementary Figure S2; Table 1). The whole-exome sequencing (WES) was conducted in July 2021, revealing that Patient 1 carried both the heterozygous c.2980delA and c.1834C>T *ABCC2* mutations. Her son also shared the heterozygous c.1834C>T *ABCC2* mutation (Figure 1B; Table 2). Considering her symptoms, clinical examinations, and genetic testing, she was diagnosed with DJS. Intrahepatic cholestasis of pregnancy (ICP) was excluded.

**TABLE 1:** Clinical laboratory tests and imaging examinations in the patients with Dubin–Johnson syndrome.

Patients number	WBC (10^9^/L)	Hb (g/L)	PLT (10^9^/L)	ALT/AST (u/l)	Alb (g/L)	TBIL/DBIL (umol/l)	TBA (umol/l)	GGT (u/l)	ALP (u/l)	Abdominal imaging examination
Patient 1	6.92	126	257	35.4/12.3	36.5	82.8/65.1	8.7	17.9	NA	Splenomegaly and hepatic hemangioma
Patient 2	3.71	155	263	17.8/18.9	46.1	110.6/70.4	3.5	NA	NA	Hepatomegaly

WBC, white blood cells (normal range: 3.5–9.5*10^9^/L); Hb, hemoglobin (normal range: 130–170 g/L); PLT, platelet (125–350*10^9^/L); ALT, alanine aminotransferase (normal range: 9–50 u/l); AST, aspartate aminotransferase (normal range:15–40 u/l); Alb, albumin (normal range: 40–50 g/L); TBIL, total bilirubin (normal range: 3.4–17.1 umol/l); DBIL, direct bilirubin (normal range: normal range: 0–6 umol/l); TBA: total bile acid (normal range: 0–10 umol/l); GGT, gamma-glutamyl transpeptidase (normal range: 35–100 u/l); NA: not available.

**TABLE 2:** Variants of *ABCC2* in the patients with Dubin–Johnson syndrome.

	Homozygous/heterozygous	Mutation	Type	Exon	Location in MRP2 protein	Origin	ACMG classification
Patient 1	Compound heterozygous	c.2980delA/p. I994Lfs*29	Deletion	Exon22	MSD3	NA	Pathogenic
		c.1834C>T/p. R612W	Missense	Exon14	Peptide chain linking MSD2 and NBD1	NA	Likely pathogenic
Patient 2	Compound heterozygous	c.2980delA/p. I994Lfs*29	Deletion	Exon22	MSD3	Mother	Pathogenic
		c.4465_4473delinsGGCCCACAG/p. I1489_I1491delinsGPQ	Deletion-insertion	Exon31	NBD2	Father	Likely pathogenic

ACMG, American college of medical genetics and genomics; MSD, membrane spanning domain; NBD, nucleotide binding domains1.

**FIGURE 2:**
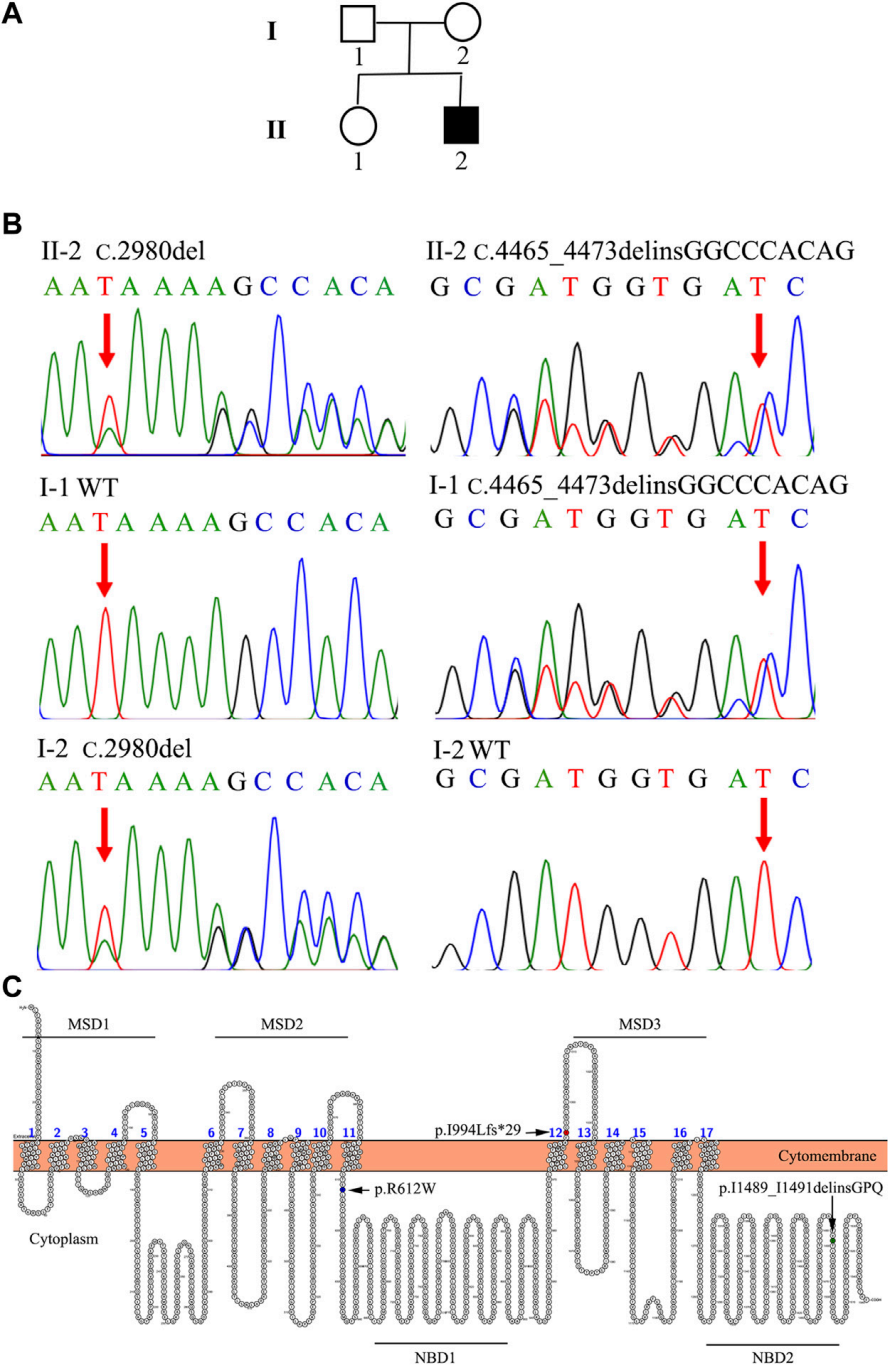
The family tree and genetic analysis of Family 2. **(A)** Proband II-2 suffered from DJS. **(B)** Direct sequencing showed a compound heterozygous (c.2980delA and c.4465_4473 delinsGGCCCACAG) *ABCC2* mutation in II-2. I-1 was affected with the heterozygous c.4465_4473delinsGGCCCACAG mutation. II-2 harbored the heterozygous c.2980delA variant. **(C)** Locations of the mutations in MRP2. The schematic diagram of MRP2 was generated using Protter software (http://wlab.ethz.ch/protter/start/). MSD: membrane spanning domains, NBD: nucleotide binding domains.

In Family 2, an 18-year-old male (Patient 2) presented with intermittent mild jaundice for more than 9 years. This patient showed intermittent jaundice from the age of nine, which would be aggravated by fatigue. He had a previously free medical history and no special personal and family history (Figure 2A). When he came as a medical genetic outpatient to our hospital, he presented with yellow skin and sclera. In the physical examination, his liver could be palpated at the lower margin of the ribs. Patient 2’s TBIL level was 110.6 μmol/L and his DBIL level was 70.4 μmol/L, with no other abnormalities in the liver function test. There were no abnormalities in autoimmune liver disease-associated antibodies or serum hepatitis B and C virus testing. A CT scan revealed hepatomegaly. The lower margin of the liver exceeded the lower costal margin (Table 1, Supplementary Figure S2). The WES indicated that Patient 2 harbored the heterozygous c.2980delA variant inherited from his mother and the heterozygous c.4465_4473delinsGGCCCACAG mutation inherited from his father (Figure 2B; Table 2). He was also diagnosed with DJS and chose to observe his jaundice (without drug interventions). In the telephone follow-up, he still complained of intermittent jaundice.

However, both patients refused the 99mTc-HIDA cholescintigraphy and liver biopsy. The candidate variants in the *ABCC2* gene were validated by Sanger sequencing. The primer sequences are listed in Table A1. The methods of genetic testing are described in the supplementary materials. The family members participating in the study signed written informed consent forms. This study complied with the ethical guidelines of the Declaration of Helsinki and was approved by the ethics committee of the Second Xiangya Hospital of Central South University (2014 ethical approval No. S046).

**TABLE A1:** Primer sequences used for mutation sequencing of *ABCC2* gene.

Mutations in *ABCC2*	Primer sequences 5'→3'
Exon 14	F: GGC​AGG​AGG​TAG​TTT​CTG​GA
	R: TGC​AGG​ATT​CCC​TGC​TTA​TC
Exon 22	F: GCA​TTG​TTC​TAT​TAT​CCT​TCC​TG
	R: ATG​CAT​GGA​CGA​AAC​CAA​AG
Exon 31	F: ATT​CCA​GGG​GAA​TTT​TGG​AG
	R: CTCACGGCTTGAGCTTCC

F, forward; R, revers.

## Discussion

The two patients suffered from chronic low-grade predominantly conjugated hyperbilirubinemia. There were no other features of hepatobiliary disorder. Patient 1’s jaundice increased during pregnancy and while taking oral contraceptives, which was in line with DJS (Erlinger et al., 2014). In the liver biopsy, specimens of DJS had a grossly black appearance and coarse, deep-brown, pigmented granules (Memon et al., 2016). The expression of MRP2 was absent on the hepatocellular membrane in patients with DJS (Morii and Yamamoto, 2016). The MRP2 transporter mediates the canalicular secretion of several organic anions, including many drugs, toxicants, and endogenous compounds (Keppler, 2011). Pigment accumulation is caused by the retention of anionic metabolites of tyrosine, phenylalanine, and tryptophan, which polymerized to form a similar melanin-like pigment in the liver (Kitamura et al., 1992). Postoperative 99mTc-HIDA cholescintigraphy would reveal delayed visualization of the extrahepatic bile ducts (Park et al., 2018). Most patients with DJS have a normal lifespan. Liu *et al.* even used a graft from a donor with DJS to conduct living donor liver transplantation. The recipient was followed for 1 year and the results were satisfactory (Liu et al., 2012). However, some drugs, such as methotrexate, vincristine, and cisplatin, are excreted from hepatocytes via the MRP2 transporter (Zhang and Fu, 2011). The mutant MRP2 transporter could affect their sensitivity and toxicity (van der Schoor et al., 2015). Therefore, adverse drug reactions need to be carefully noted for DJS patients.

The c.2980delA was a frameshift variant in the *ABCC2* gene. Loss of function is a known mechanism of DJS (PVS1). In addition, the c.2980delA was absent from the controls in the 1000G and EXAC databases (PM2). The use of the MutationTaster prediction software (Schwarz et al., 2014) revealed a deleterious effect from c.2980delA (PP3). Patient 1’s phenotype was highly specific for DJS (PP4). Considering the ACMG guidance (Richards et al., 2015), we classified the c.2980delA variant as a pathogenic mutation.

The c.1834C>T mutation was not found in the 1000G or EXAC database (PM2). In addition, c.1834C>T was detected in trans with the pathogenic variant (c.2980delA) in Patient 1, which was confirmed by testing the offspring of her son (PM3). In addition, MutationTaster, PolyPhen-2 (Adzhubei et al., 2010), and SIFT (Kumar et al., 2009) predicted a disease-causing effect for the mutation (PP3). Patient 1 showed highly specific symptoms for DJS (PP4). Based on the ACMG guidelines, c.1834C>T was a likely pathogenic variant. The term “likely pathogenic” indicates a greater than 90% certainty of a variant being disease-causing. The variant had been recorded by gnomAD (with an allele frequency of 4.065e-06) and InterVar (with Uncertain clinical significance) database. But it was clarified for the first time as a likely pathogenic mutation associated with DJS.

The c.2980delA variant was also found in Patient 2. It was a pathogenic variant as analyzed above. The other mutation, c.4465_4473delinsGGCCCACAG, was not found in the 1000G or EXAC database (PM2). For a recessive disorder, c.4465_4473delinsGGCCCACAG was detected in trans with a pathogenic variant (c.2980delA), which was confirmed in his parents (PM3). The c.2980delA and c.4465_4473delinsGGCCCACAG formed a compound heterozygous mutation in Patient 2. The compound heterozygous variant was found to cosegregate in the family members (PP1). In addition, multiple lines of computational evidence supported a deleterious effect of the gene (PP3). Patient 2’s phenotype was highly specific for DJS (PP4). Therefore, c.4465_4473delinsGGCCCACAG was identified as a likely pathogenic variant according to the ACMG guidelines. By scanning the HGMD and previously published works, we concluded that the three mutations are novel pathogenic variants.

Beside the three *ABCC2* mutations identified during the steps in the filtering strategies in the WES-yielded data, we also detected a heterozygous mutation, p. G71R, in the *UGT1A1* gene in Patient 1 (Table A2). *UGT1A1* is the disease-causing gene of Gilbert syndrome (GS), Criggler–Najjar syndrome I (CNS-I), and Criggler–Najjar syndrome II (CNS-II). However, the three diseases are autosomal recessive diseases and cause predominantly unconjugated hyperbilirubinemia. This is not consistent with the patient’s symptoms. In addition, the minor allele frequency of the variant is greater than 0.01 in the 1000G, ExAC, and gnomAD databases. Therefore, we excluded the variant. DJS and Rotor syndrome have similar symptoms, but no pathogenic variant was detected in the *OATP1B1* or *OATP1B3* genes, which are causative genes for Rotor syndrome. Therefore, Rotor syndrome was excluded. Furthermore, there were no candidate mutations in the *ATP8B1, ABCB4*, or *ABCB11* genes, which could cause benign recurrent intrahepatic cholestasis (BRIC) or ICP.

**TABLE A2:** Other bilirubin-metabolism related mutation in the patients

Patient	Gene	Mutation	Exon	Related diseases
Patient 1	*UGT1A1* (NM_000463)	c.211G>A, p.G71R, g.234669144G>A (heterozygous)	Exon 1	GS, CNS-I, CNS-II

GS, Gilbert syndrome; CNS-I, Criggler–Najjar syndrome I; CNS-II, Criggler–Najjar syndrome

MRP2 consists of three membrane spanning domains (MSDs) which include 17 transmembrane helices and two intracellular highly conserved nucleotide binding domains (NBDs). The NBD has an ATP binding site and is important for transporting some organic anions out of the hepatocyte. The transport process by MRP2 relies on the energy generated by the hydrolysis of ATP. There have been studies on genotype-phenotype correlations in DJS (Lee et al., 2006; Togawa et al., 2018; Kim et al., 2020). Mutations located in the NBD domain have been associated with early-onset DJS (Lee et al., 2006). Our two patients carried the same heterozygous c.2980delA/p.I994Lfs*29 mutation in the MSD3 domain. However, Patient 2 was affected by a heterozygous deletion-insertion mutation—c.4465_4473delinsGGCCCACAG/p.I1489_I1491delinsGPQ—in the NBD2 domain. Patient 1 carried a heterozygous missense mutation—c.1834C>T/p.R612W—in the peptide chain linking MSD2 and NBD1 (Wen et al., 2017; Xiang et al., 2017) (Figure 2C). Patient 2 carried the variant in the NBD2 domain. He suffered from early-onset and more serious hyperbilirubinemia than Patient 1, consistent with the above study. In addition, the combination of missense and truncating variants may be essential for the phenotype of neonatal DJS (Togawa et al., 2018). Patient 1 was affected by missense and truncating variants but she did not show neonatal DJS.

Because of the favorable prognosis of DJS, the two patients’ stresses were significantly relieved. Patient 1 could also avoid using oral contraceptives, which might aggravate her jaundice. DJS could not cause splenomegaly. The etiology of splenomegaly requires further analysis and follow-up. Patient 2 believed that the genetic testing could facilitate his search for work, as an unclear diagnosis of his liver disease would restrict the range of his job choices. Both patients were satisfied with the outcome of the genetic testing. However, there were several limitations in our research. The 99mTc-HIDA cholescintigraphy and biopsy could not be conducted because of the patients’ rejections. In addition, functional experiments of the novel pathogenic *ABCC2* mutations need further investigation.

In conclusion, this study identified three novel pathogenic *ABCC2* mutations in two patients with DJS. These results contribute to the genetic diagnosis and counseling of families with DJS and expand the spectrum of *ABCC2* mutations.

## Data Availability

The datasets for this article are not publicly available due to concerns regarding participant/patient anonymity. Requests to access the datasets should be directed to the corresponding author.

## Supplementary material

Supplementary Figure S1
**(A)** Ttimelines with relevant data from Patient 1. She was tested for hyperbilirubinemia in 2015 without obvious symptoms. She fell pregnant in November 2017 and, from then on, her jaundice gradually aggravated. The jaundice was in remission after her parturition. However, the hyperbilirubinemia deteriorated again after she took oral contraceptives. She finally received genetic testing in July 2021. **(B)** The bilirubin concentrations were during Patient 1’s pregnancy and drug interventions. She gave birth on 18 August 2018. The drug interventions were conducted from 16 August 2018 to 31 August 2018. TBIL: total bilirubin, DBIL: direct bilirubin.

Supplementary Figure S2 The image data of the patients. **(A-C) **The results of Patient 1's ultrasound examinations. **(A)** The thickness of her spleen was about 49 mm. **(B)** The inferior margin of the spleen was about 20 mm below the costal margin. **(C)** The hemangioma in the liver (about 7.4 mm*7.6 mm). **(D**) The CT scan showed that Patient 2 was affected by hepatomegaly. The left lobe of the liver extended to the lower left.

## AppendixGenetic testing methods.

The genomic DNA was isolated from peripheral blood using a Blood gDNA Miniprep Kit (Hangzhou Beiwo Meditech Co., Ltd., Hangzhou, China). The WES was conducted on Patients 1 and 2, which was performed by AmCare Genomics Lab (Guangzhou, China). High-throughput sequencing was performed on the MGISEQ-2000 platform (MGI Tech Co., Ltd., Shenzhen, China). Basic bioinformatics analysis, including read, mapping and variant detection, was also completed by AmCare Genomics Lab Limited.

The methods of WES data filtering were as follows: (1) We excluded variants in intergenic, intronic, and untranslated regions (UTRs) and synonymous variants. (2) Variants in the databases with a minor allele frequency>0.01 were excluded. The databases included the 1000 Genomes project (1000G; http://browser.1000genomes.org/), Exome Aggregation Consortium (ExAC; http://exac.broadinstitute.org/), Exome Sequencing Project 6500 (esp6500; http://evs.gs.washington.edu/EVS/), and Genome Aggregation (gnomAD; http://gnomad.broadinstitute.org/). (3) The candidate pathogenetic variants in bilirubin-metabolism related genes were retained. (4) The pathogenicity of the variants was analyzed according to the American College of Medical Genetics and Genomics (ACMG) guidance for the interpretation of sequence variants.

The potential variants from WES were validated by Sanger sequencing. The primer sequences were listed in Table A1. PCR products were sequenced on a Superyears Classic Genetic Analyzer (Nanjing Superyears Gene Technology Co., Ltd., Nanjing, China).
