# Supplementary figures and images for: Case Report: Three novel pathogenic ABCC2 mutations identified in two patients with Dubin–Johnson syndrome
Source: Front Genet. 2022 Aug 25;13:895247. doi: 10.3389/fgene.2022.895247 (PMC9452728; doi:10.3389/fgene.2022.895247)

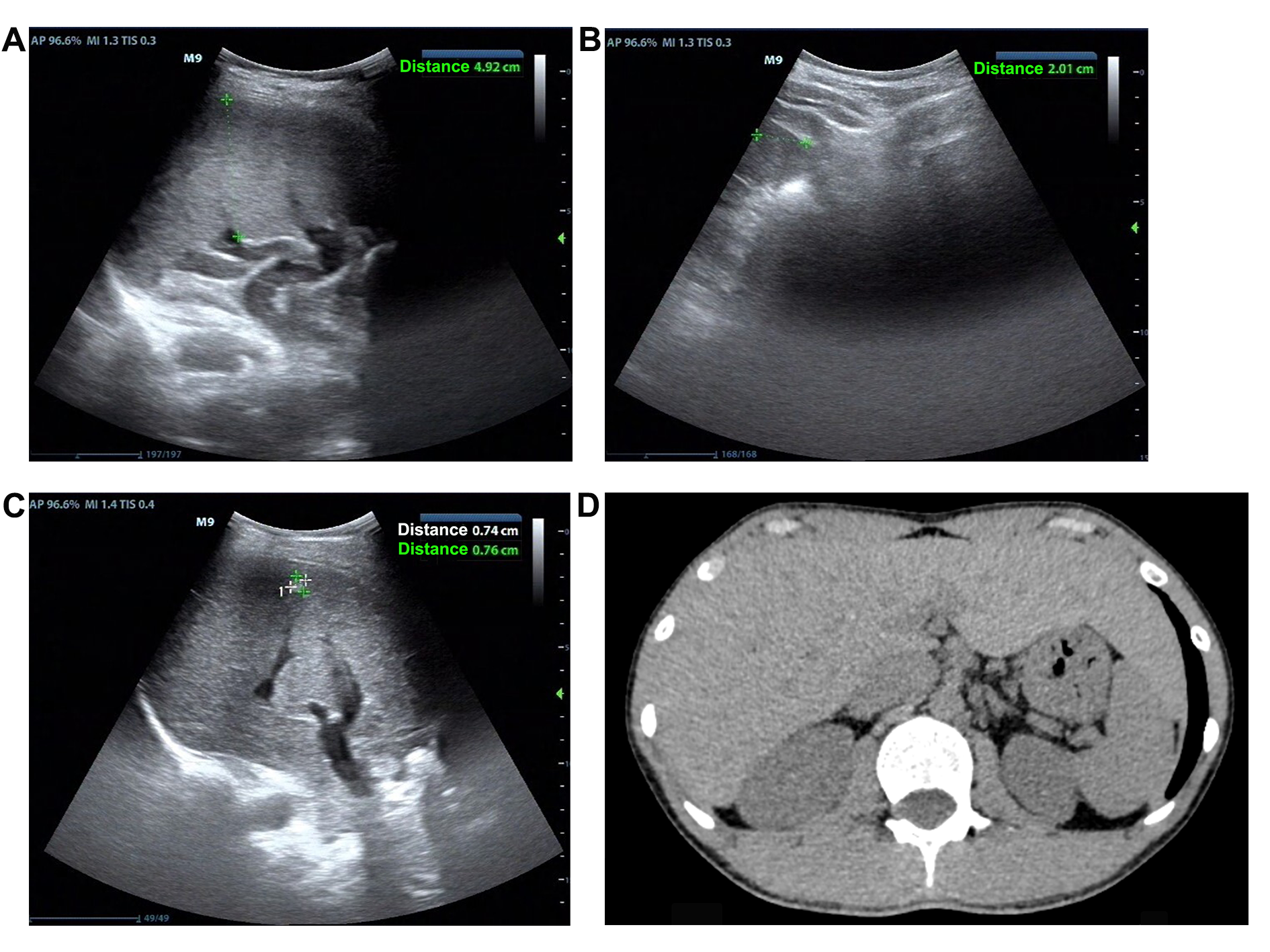

Supplement: Supplementary file 1 [file Image2.TIF]

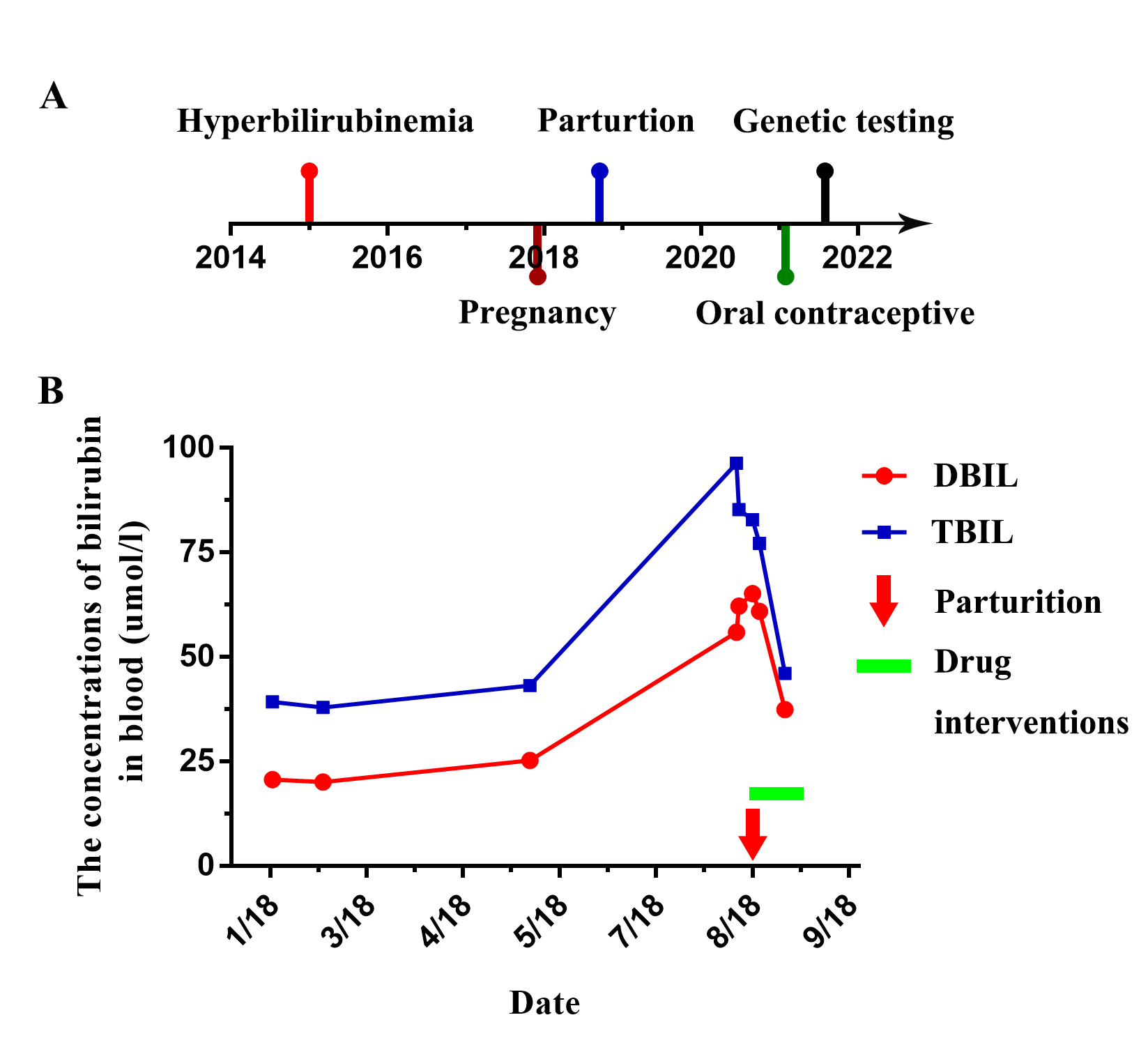

Supplement: Supplementary file 2 [file Image1.TIF]
